# Preconception paternal alcohol exposure exerts sex-specific effects on offspring growth and long-term metabolic programming

**DOI:** 10.1186/s13072-019-0254-0

**Published:** 2019-01-22

**Authors:** Richard C. Chang, Haiqing Wang, Yudhishtar Bedi, Michael C. Golding

**Affiliations:** Department of Veterinary Physiology and Pharmacology, College of Veterinary Medicine and Biomedical Sciences, Texas A&M University, College Station, TX 77843-4466 USA

**Keywords:** Paternal alcohol use, Metabolic programming, Preconception exposure, Epigenetic programming, Developmental origins of adult disease, Growth restriction, Paternal exposure, Epigenetics, Fetal alcohol spectrum disorder

## Abstract

**Background:**

Although clinical data support an association between paternal alcohol use and deficits in child neurocognitive development, the relationship between paternal drinking and alcohol-induced growth phenotypes remains challenging to define. Using an established mouse model of chronic exposure, previous work by our group has linked preconception paternal alcohol use to sex-specific patterns of fetal growth restriction and placental dysfunction. The aim of the present study was to investigate the long-term impact of chronic preconception paternal alcohol use on offspring growth and metabolic programming.

**Results:**

Preconception paternal alcohol exposure induced a prolonged period of fetal gestation and an increased incidence of intrauterine growth restriction, which affected the male offspring to a greater extent than the females. While the female offspring of ethanol-exposed males were able to match the body weights of the controls within the first 2 weeks of postnatal life, male offspring continued to display an 11% reduction in weight at 5 weeks of age and a 6% reduction at 8 weeks of age. The observed growth deficits associated with insulin hypersensitivity in the male offspring, while in contrast, females displayed a modest lag in their glucose tolerance test. These metabolic defects were associated with an up-regulation of genes within the pro-fibrotic *TGF*-*β* signaling pathway and increased levels of cellular hydroxyproline within the livers of the male offspring. We observed suppressed cytokine profiles within the liver and pancreas of both the male and female offspring, which correlated with the up-regulation of genes in the LiverX/RetinoidX/FarnesoidX receptor pathways. However, patterns of gene expression were highly variable between the offspring of alcohol-exposed sires. In the adult offspring of alcohol-exposed males, we did not observe any differences in the allelic expression of *Igf2* or any other imprinted genes.

**Conclusions:**

The impact of paternal alcohol use on child development is poorly explored and represents a significant gap in our understanding of the teratogenic effects of ethanol. Our studies implicate paternal exposure history as an additional and important modifier of alcohol-induced growth phenotypes and challenge the current maternal-centric exposure paradigm.

**Electronic supplementary material:**

The online version of this article (10.1186/s13072-019-0254-0) contains supplementary material, which is available to authorized users.

## Background

In clinical studies, fetal alcohol spectrum disorders (FASDs) associate with three broad developmental defects: distinctive craniofacial malformations, central nervous system defects and both prenatal and postnatal growth restriction [1, 2]. The growth defects are characterized by reductions in height, weight, and body mass index that manifest at birth and continue to persist through young adulthood [3–5]. Indeed, although perceived as a childhood disorder, the growth phenotypes associated with FASDs are lifelong, with long-term growth restriction, as well as immune dysfunction, hyperinsulinemia, and other endocrine disruptions persisting into adulthood [5–10]. As a consequence of these persistent abnormalities, the life expectancy of patients with fetal alcohol syndrome is 34 years, which is 58% lower than the general population [11]. This dramatic reduction is very likely linked to the capacity of alcohol to significantly alter developmental programming, which promotes the early onset of adult disease [12, 13]. However, while much research has focused on the neurological phenotypes of FASDs, the relationship between ethanol exposures and the long-term effects on growth and metabolic programming has received comparatively little attention.

FASD growth phenotypes can be linked to alcohol-induced intrauterine growth restriction mediated by impaired placentation [4, 14–17]. Impaired placentation, in turn, associates with poor cognitive development and long-term alterations in metabolic programming within the offspring [18–25]. Thus, the long-term effects of ethanol on growth and adult health may be linked to early developmental insults arising from a compromised fetal–maternal interface. Importantly, emerging research can now link a number of preconception exposures to compromised placentation and long-term alterations in metabolic function within the offspring [26–28]. These data further emphasize the importance parental histories of drug use, social stress, and environmental exposures have on child health and may help explain the enormous variation observed in FASD phenotypes and incidence [29].

The impacts parental histories of alcohol use have on child development are poorly explored and represent a significant gap in our understanding of the teratogenic potential of ethanol. This is especially true of preconception male alcohol exposures, which are a largely ignored aspect of patient history and are similarly underexplored in relevant biomedical models [30]. To this point, emerging epidemiological research indicates preconception paternal alcohol exposures have the ability to significantly influence child health and development [31, 32]. For example, clinical data can correlate paternal alcoholism with negative impacts on child behavior [33–38] and cognitive development [39–43]. In addition, clinical associations between paternal drinking and increased rates of congenital abnormalities, as well as decreases in infant birth weight and head circumference, have been reported [44–46]. However, in these studies, additional and often uncontrolled factors such as nutrition, poor housing conditions, maternal stress, smoking, and parental alcohol use all exert independent effects on child growth and development. In this setting, it is virtually impossible to identify a direct link between preconception paternal alcohol use and child development [47, 48].

In rodent models, preconception paternal alcohol exposures associate with reductions in birth weight, increased incidences of congenital anomalies and cognitive impairment in the offspring [31, 49–58]. These observations join a growing body of work demonstrating the capacity of multiple stressors to alter the sperm-inherited developmental program [59–78] and suggest preconception paternal alcohol exposures may exert a long-term influence on offspring growth and development. In rodent models of maternal exposure, alcohol-induced abnormalities in the germline programming of the hypothalamic-pituitary stress response transmit through multiple generations via the male germline but are not maternally inherited [79]. Thus, alcohol has the ability to exert a lasting impact on the male-inherited developmental program, which, similar to studies employing models of intrauterine ethanol exposure [80–85], may exert long-term impacts on offspring health. However, no studies have yet directly examined the associations between paternal preconception alcohol exposures and long-term growth and metabolic programming within the offspring.

Recent work by our group has linked chronic preconception male alcohol exposure to fetal abnormalities in cholesterol trafficking, sex-specific patterns of growth restriction, disruptions in the regulation of imprinted genes and alterations in the genetic pathways regulating hepatic fibrosis [58]. The identification of altered cholesterol trafficking and increased markers of hepatic fibrosis within the fetus raise the prospect that these paternally inherited abnormalities have the capacity to significantly impact the long-term metabolic function of the offspring. This notion is further reinforced by the identification of compromised placental function in the offspring of alcohol-exposed males [58]. On the basis of these considerations, the aim of the present study was to investigate the long-term impact of chronic preconception paternal alcohol exposure on offspring growth and metabolic programming.

## Materials and methods

### Animal work

All experiments were conducted under AUP 2017-0308 and approved by the Texas A&M University IACUC. In the outlined experiments, the male mice were of a C57BL/6(Cast7) background, while female mice were C57BL/6J (RRID:IMSR_JAX:000664). The C57BL/6(Cast7) strain of mice were generated in the Bartolomei laboratory and were selected to possess portions of a *Mus* m*usculus castaneus* chromosome 7 and chromosome 12 (where at least 5 imprinting domains and more than 30 imprinted genes reside) bred onto a C57BL/6J background [86]. When using crosses between the B6(Cast7) strain and a C57BL/6J strain, we can distinguish the maternal and paternal alleles of select genes using C57BL/6(Cast7) and C57BL/6J polymorphisms that we have identified previously [58, 87]. The C57BL/6(Cast7) strain of mice is on a C57BL/6J background, which is susceptible to alcohol-induced teratogenesis [88, 89].

To investigate the long-term impact of alcohol exposure on the male-inherited developmental program, an established and well-characterized mouse model of chronic alcohol exposure was employed [90]. Here, postnatal day 90, adult males were provided limited access to ethanol during a 4-h window of the night cycle. This rodent model (Drinking in the Dark) promotes the voluntary consumption of ethanol in sufficient quantities to achieve pharmacologically meaningful blood alcohol concentrations, typically in excess of 150 mg/dL [58, 91]. In a study examining the drinking patterns of 10,424 college freshmen in the USA, one in five males reported routinely consuming 10 + drinks in a single session, while half reported drinking beyond the binge level (5 + drinks in a single session) [92, 93]. Thus, the blood alcohol levels observed in our model reflect a range frequently obtained by college age males [92–94].

Here, individually caged, postnatal day 90, adult males were fed a standard diet (catalog# 2019, Teklad Diets, Madison, WI, USA) and maintained on a 12-h light/dark cycle. Males were provided limited access to ethanol during a 4-h window, beginning 1 h after the initiation of the dark cycle [58]. During this 4-h window, experimental males were provided access to a solution of 10% (w/v) ethanol (catalog# E7023; Millipore-Sigma, St. Louis, MO, USA) and 0.066% (w/v) Sweet’N Low (Cumberland Packing Corp, Brooklyn NY, USA), while control males received a solution of 0.066% (w/v) Sweet’N Low alone. After each session, the amount of fluid consumed by each mouse was recorded.

The addition of Sweet’N Low is necessary to encourage male mice to develop consistent drinking habits. Although, prolonged exposure to a 10% Sweet’N Low solution has previously been shown to drive the development of glucose intolerance through functional alterations to the intestinal microbiota, the concentrations employed in our studies were 150-fold lower than those utilized in these previous experiments [95]. In addition, we were careful to ensure that mice in both preconception treatment groups consumed equivalent fluid volumes and therefore received identical exposures to Sweet’N Low.

Once consistent patterns of drinking were established, males were maintained on this protocol for a period of 70 days, which corresponds to the length of approximately two complete spermatogenic cycles, thereby ensuring that both pre-meiotic and post-meiotic spermatids were exposed to alcohol [96, 97]. Once the 70-day preconception treatment was achieved, two naturally cycling females were placed into a new cage along with each exposed male. During these matings, males were not provided access to the alcohol/control preconception treatments. The next morning, matings were confirmed by the presence of a vaginal plug and both the male and female mice were returned to their original cages. Males were allowed a 24-h rest period, during which the preconception exposure was resumed and then used in a subsequent mating. This procedure was repeated until each male had produced a minimum of three litters.

Pregnant dams were maintained on a breeder diet (catalog# 5058; LabDiet, St. Louis, MO, USA), subjected to minimal handling and monitored for delivery twice daily. One week after birth, fifteen male and female offspring from each preconception treatment group were randomly selected from across at least five different litters and monitored for postnatal growth and development. Mice were maintained on a standard diet (catalog# 2019; Teklad Diets, Madison, WI, USA) and body weight was recorded weekly, for 8 weeks. Between seven and 8 weeks of age, metabolic function was assayed using glucose and insulin tolerance tests. After 8 weeks of age, offspring were terminated and both physiological fluids and tissues were collected.

### Insulin and glucose tolerance tests

Beginning at 7 weeks of age, mice were fasted overnight for 12 h and tested for glucose and insulin tolerance, with a minimum of a 1-week recovery time between these separate tests. Here, mice received a single intraperitoneal injection of d-glucose (2 g/kg body weight; catalog# SG8270; Millipore-Sigma, St. Louis, MO, USA) or insulin (1 unit/kg body weight; catalog# 89508-914; VWR, Radnor, PA, USA) and blood glucose levels measured using Clarity Plus-Blood Glucose Test Strips (catalog# DTG-GL5PLUS; Clarity Diagnostics, Boca Raton, Florida USA) from 5 μL of blood drawn from the tail vein. For glucose tolerance tests, blood glucose levels were measured before the injection of glucose and at 30, 60, 90 and 120 min post-injection. For insulin tolerance tests, blood glucose levels were measured before the injection of insulin and at 15, 30, 45 and 60 min after insulin injection. Each experimental group contains fifteen male and female animals (*n* = 15).

### Liver perfusion assay

One week after the final evaluation of metabolic parameters, six males and six females from each group were randomly selected to evaluate insulin signaling and levels of AKT phosphorylation (Ser473) in the liver. Mice were fasted 12 h overnight and anesthetized with 2% isoflurane [98], until animals achieved a deep plane of anesthesia, demonstrating a lack of a righting reflex and a ~ 50% reduction in respiratory rate. At this point, the body cavity of the mouse was opened and the liver perfused with either 37 °C PBS + 0.1% BSA (Control) or 2 units/kg of an insulin solution (10 nM insulin, Sigma, in PBS + 0.1% BSA; catalog# 89508-914; VWR, Radnor, PA, USA) at a flow rate of 100 ml/h. Here, the suprahepatic vessel was clamped and a 27-gauge syringe inserted into the intrahepatic cava. After observing liver perfusion, the hepatic portal vein was cut open. During this procedure, a nose cone containing gauze soaked in isoflurane was kept over the nose of the animal and removed only when the rate of respiration dipped down below 25%. After 5 min, animals were euthanized by cervical dislocation and tissues of interest, including liver, kidney and pancreas collected and snap-frozen in liquid nitrogen. Each experimental group contains six different animals (*n* = 6).

### Western immunoblot analysis

Liver tissue samples were collected and homogenized in a Tris lysis buffer including 50 mM Tris, 1 mM EGTA, 150 mM NaCl, 1% Triton X-100, 1% β-mercaptoethanol, 50 mM NaF, 1 mM Na3VO4 and pH 7.5. Samples were separated on 10% sodium dodecyl sulfate–polyacrylamide gels by electrophoresis and transferred to nitrocellulose membranes. The primary antibodies used in this study were as follows: *anti*-*phosphorylated protein kinase B* (*AKT*) at Serine 473 (catalog#700392; RRID:AB_2532320; Thermo-Fisher, Waltham, MA, USA) and anti-*AKT* used for loading control (catalog#44609G; RRID:AB_2533692; Thermo-Fisher, Waltham, MA, USA). Blots were visualized by using secondary antibodies conjugated to horseradish peroxidase (catalog# sc-2004; RRID:AB_631746; Santa Cruz Biotechnology, Santa Cruz, CA, USA) and an enhanced chemiluminescence detection system (Pierce, Rockford, IL, USA). Relative *AKT* phosphorylation was derived as a ratio to total *AKT*. Band intensities were quantified by densitometry using ImageJ (RRID:SCR_003070; National Institutes of Health, Bethesda, MD, USA). Each experimental group contains six different animals (*n* = 6).

### Measurement of physiological parameters

Male plasma alcohol concentrations were measured using an Ethanol Assay Kit (catalog# ECET100; BioAssay Systems, Hayward, CA, USA) according to manufacturer’s protocol. Animals were euthanized by cervical dislocation, blood collected postmortem and plasma insulin levels determined using the Mouse Insulin ELISA kit (catalog# EMINS; Thermo-Fisher, Waltham, MA, USA), according to the recommended protocol. Levels of *IL1B*, *IL6*, *INFg* and *TNFa* were determined using commercial ELISA assays (catalog# KMC0061 and KMC0011; Thermo-Fisher, Waltham, MA, USA and catalog# Ab100689 and Ab100747; Abcam, Cambridge, MA, USA). Total cholesterol levels were determined using the Total Cholesterol Assay Kit (catalog# STA-384; Cell Biolab, Inc, San Diego, CA USA), according to the recommended protocol. The comparative levels of low-density and high-density lipoproteins were determined using a Cholesterol Assay Kit (catalog# ab65390; Abcam, Cambridge, MA, USA), according to the recommended protocol. The levels of hydroxyproline were determined using the Hydroxyproline Assay Kit (catalog# MAK008; Millipore-Sigma, St. Louis, MO, USA), following to the recommended protocol.

### RNA analyses

Total RNA was isolated from 8-week-old offspring liver using the RNeasy Plus Mini Kit, (catalog# 74134; Qiagen, Germantown MD, USA) according to manufacturer’s instructions. Samples were randomized prior to RNA-seq library preparation. Libraries were generated from 10 ng of RNA using the TruSeq RNA Sample Preparation kit (Illumina, San Diego, CA, USA) and pooled for sequencing on an Illumina HiSeq 2500 at the Whitehead Genome Technology Core (Cambridge, MA, USA). Sequencing data were demultiplexed and aligned using STAR (RRID:SCR_015899) with default parameters [99].

### RNA deep sequencing data analysis, selection of candidate mRNAs and functional enrichment

Following deep sequencing, Bowtie (RRID:SCR_005476) and Tophat (RRID:SCR_013035) were used to align 50-bp-length, paired-end reads into transcripts using the *Mus musculus* (UCSC version mm10) reference genome. To measure the relative abundance of each transcript, the resulting aligned reads were analyzed using the Cufflinks suite (RRID:SCR_014597). Expression was quantified as the number of reads mapping to a gene divided by the gene length in kilobases and the total number of mapped reads in millions and designated as fragments per kilobase of exon per million fragments mapped (FPKM). To select differentially expressed transcripts, a volcano plot measuring statistical significance and magnitude of fold change was generated based on the log2 fold change (Y-axis) and − log10 *p* value from Cuffdiff analysis within the Cufflinks suite (RRID:SCR_014597) (X-axis). Differentially expressed mRNAs were selected on the basis of linear p-value cutoff of 0.05, which was considered significant and highlighted by colored dots in the volcano plot. Subsequently, functional clusters were identified by applying Ingenuity Pathway Analysis (RRID:SCR_008653; Ingenuity System Inc, Redwood City, CA, USA) [100].

### Real-time RT-qPCR analysis of gene expression

Total RNA was isolated from 8-week liver using the RNeasy Plus Mini Kit, (catalog# 74134; Qiagen, Germantown, MD, USA) according to manufacturer’s instructions. One microgram of purified total RNA was treated with amplification grade DNase I (catalog# AMPD1; Millipore-Sigma, St. Louis, MO, USA) according to the manufacturer’s recommendations and seeded into a reverse transcription reaction using the High Capacity cDNA Reverse Transcription Kit (catalog# 4368814; Thermo-Fisher, Waltham, MA, USA), where the reaction mixture was brought to 25 °C for 10 min, 37 °C for 120 min and then 70 °C for 5 min. Relative levels of candidate gene transcripts were analyzed using the Dynamo Flash mastermix (catalog# F-415XL; Thermo-Fisher, Waltham, MA, USA) according to the recommended protocol. Reactions were performed on a Bio-Rad CFX384. Primer sequences are listed in Additional file 1.

### Data handling and statistical analysis

For all experiments, measures were input into the statistical analysis program GraphPad (RRID:SCR_002798; GraphPad Software, Inc., La Jolla, CA, USA), and statistical significance was set at alpha = 0.05. All datasets were first verified for normality using the Brown-Forsythe test. In this study, the effect of two independent variables (sex vs. preconception treatment) was assessed using an analysis of variance test (ANOVA), and differences among the means evaluated using Sidak’s post hoc test of contrast. No interactions (*p* > 0.05) were observed between fetal weight and sire weight, fetal weight and dam weight, or litter size and fetal weight. Pups were defined as intrauterine growth restricted (IUGR) if their weight at 1 week of age was less than the average weight of the control group less two standard deviations [101]. The IUGR ratio was calculated by dividing the number of IUGR offspring by the total number of fetuses in each litter. To determine the significance of IUGR ratio between groups, the IUGR ratio was first transformed by arcsine square root prior to unpaired *t* test examination. The offspring were weighed weekly from week 1 through week 8. The average growth rate was calculated using the following equation: [(bodyweight at week 8 − bodyweight at week 1)/bodyweight at week 8].

For analysis of gene expression, the replicate cycle threshold (Ct) values for each transcript were compiled and normalized to the geometric mean of three validated reference genes. Transcripts encoding *tyrosine 3*-*monooxygenase/tryptophan 5*-*monooxygenase activation protein zeta* (*Ywhaz*—NM_011740), *mitochondrial ribosomal protein L1* (*Mrpl1*—NM_053158) and *hypoxanthine*-*phosphoribosyl transferase* (*Hprt*—NM_013556) were measured as reference genes. Each of these reference genes were validated for stability across treatment groups using methods described previously [102]. Normalized expression levels were calculated using the ddCt method described previously [103]. Relative fold change values from each biological replicate were determined and transferred into the statistical analysis program GraphPad. For comparisons including sex and preconception treatments, an analysis of variance (ANOVA) was utilized and Sidak’s analysis applied to comparisons with *p* values < 0.05. For single comparisons, an unpaired student’s *t* test was applied. In all instances, we have marked statistically significant differences with an asterisk.

In our mouse model, distinct single nucleotide polymorphisms between the maternal (C57BL/6J) and paternal (C57BL/6(Cast7) strains [86] allowed us to track allelic patterns of gene transcription for multiple imprinted genes. For RNA sequence-based comparisons of allelic patterns of imprinted gene expression, the proportion of single nucleotide polymorphisms was measured using the Integrative Genome Viewer (RRID:SCR_011793) software package and analyzed using either Chi-squared analysis or, if read counts were less than 5, a Fisher’s Exact test.

## Results

### Chronic paternal ethanol exposure associates with delayed parturition and intrauterine growth restriction of the offspring

**Fig. 1:**
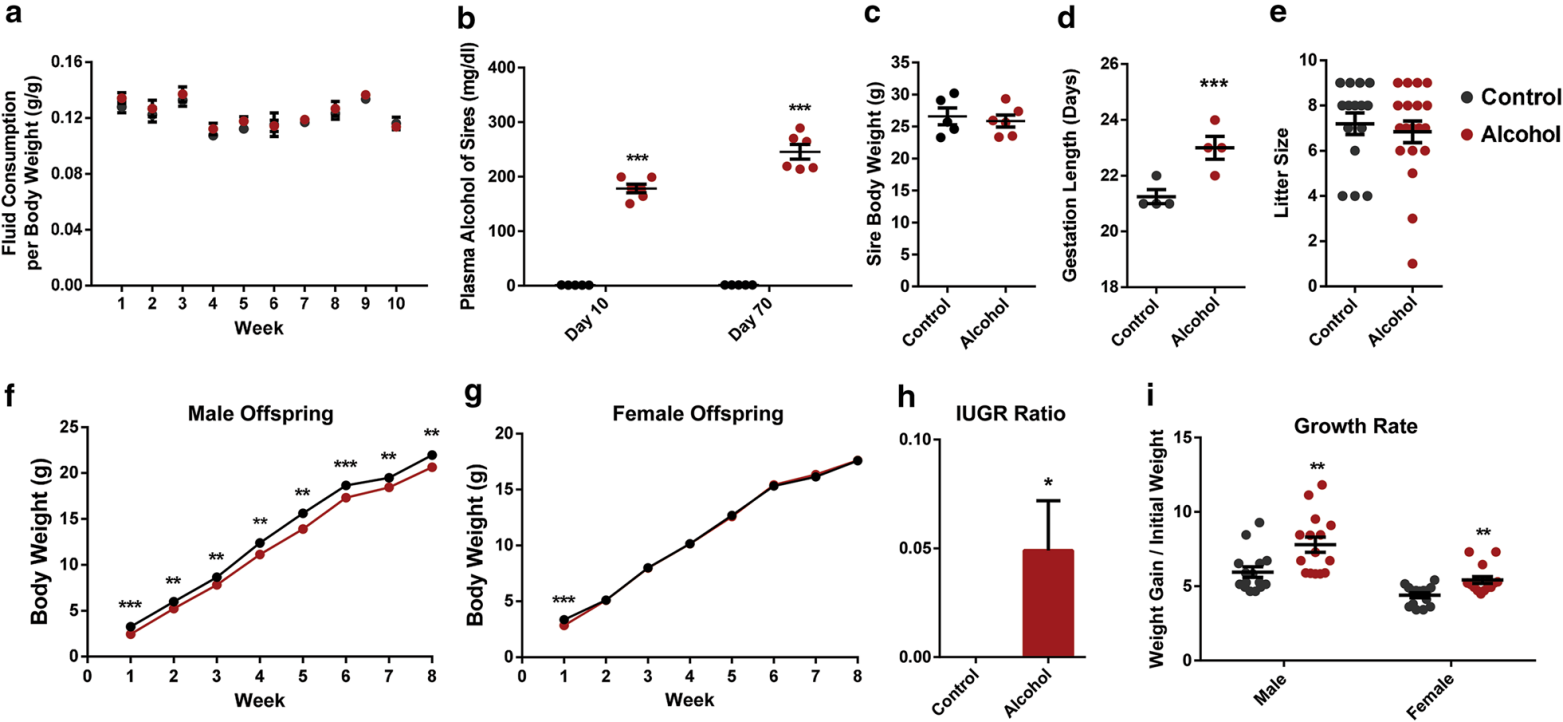
Preconception paternal ethanol exposure associates with intrauterine growth restriction (IUGR) and altered postnatal growth. **a** Average fluid consumption normalized by body weight during the preconception treatment period (*n* = 5 control and 6 experimental males). **b** Plasma alcohol concentrations measured at Day 10 and Day 70 of the 70-day preconception exposure period (*n* = 5 control and 6 alcohol-exposed males). Blood alcohol levels were measured 2 h into the 4-h exposure period. **c** Average body weight of sires at the end of the 70-day preconception exposure period (*n* = five control and six experimental males). **d** Average length of gestation for litters sired by alcohol-exposed and control males (*n* = 4). **e** Average litter size between the preconception ethanol and control treatment groups (*n* = 15 control 19 alcohol). Postnatal weights of **f** male and **g** female offspring sired by ethanol-exposed and control males measured over 8 weeks (*n* = 15 males, 15 females from each treatment group). **h** Average rate of IUGR at 1 week of age. The graph depicts the number of IUGR pups/the total number of offspring counted in each group. **i** Rate of bodyweight gain over 8 weeks of postnatal life. Error bars represent SEM *p < 0.05, **p < 0.01 and ***p < 0.001 [comparisons between 10% (w/v) ethanol plus 0.066% (w/v) Sweet’N Low (alcohol) vs. 0.066% (w/v) Sweet’N Low alone (control) preconception treatments]. Data were analyzed using either an unpaired *t* test, arcsine transformed and an unpaired *t* test with Welch’s correction applied or used in a two-way ANOVA followed by Sidak post hoc analysis

In clinical studies, FASDs are characterized by reductions in height, weight and body mass index that manifest at birth and persist through young adulthood [3, 5]. However, no studies have determined the capacity of chronic preconception male alcohol exposures to contribute to these phenotypes or impact postnatal growth. To address this gap, adult male mice were exposed to ethanol every day for a 70-day period, using a previously described limited access model [90]. Similar to our previous studies [58], no differences in fluid consumption were observed between the ethanol and control preconception treatment groups (Fig. 1a). Ethanol exposures were measured on day 10 and again on day 70 of the preconception treatment course, and in the ethanol-exposed males, it yielded average plasma alcohol levels of 178 and 245 mg/dL, respectively (Fig. 1b). During the course of the 70-day preconception treatments, no differences in weight gain could be detected between ethanol-exposed or control males (Fig. 1c). After 70-days of preconception treatment, exposed males were mated to six- to eight-week-old females. At no point during these experiments were the females ever exposed to the preconception treatments.

Litters from each of the preconception control and ethanol-exposed treatment groups were termed and the length of gestation recorded. Unexpectedly, preconception alcohol exposure associated with a 10% increase in the length of gestation (*p* < 0.05), with litters sired by ethanol-exposed males born on day 23 versus day 21 for the control litters (Fig. 1d). No differences in litter size were observed between the preconception treatment groups (Fig. 1e). At 1 week of age, paternal alcohol exposure associated with a 25% reduction in the body weight of male offspring and a 15% reduction in the body weights of the female offspring (Fig. 1f, g, *p* = 0.004, *p* = 0.017). Accordingly, the ratio of offspring displaying intrauterine growth restriction [101] was significantly increased in the offspring of ethanol-exposed males (Fig. 1h, *p* = 0.04). To determine the long-term impact of the observed growth restriction, fifteen male and fifteen female offspring were randomly selected from across five different litters and their weights tracked for 8 weeks. Interestingly, while the female offspring of ethanol-exposed males were able to match the body weights of the controls within the first 2 weeks of life, male offspring sired by alcohol-exposed fathers continued to display an 11% reduction in weight at 5 weeks of age (*p* = 0.005) and a 6% reduction at 8 weeks of age (*p* = 0.0003, Fig. 1f, g). To determine whether this difference was associated with altered growth parameters, the average growth rates for each group were calculated. Similar to clinical studies of IUGR [104], both the male and female offspring of alcohol-exposed males displayed accelerated postnatal weight gain compared to the controls (Fig. 1i). However, as in clinical reports of FASD children [5], the male offspring of ethanol-exposed fathers remained smaller than the offspring of the controls. For clarity, Additional file 2 contains the data from Fig. 1 presented in table form, with the number of replicates, statistical significance and formula used to derive each measure presented. No significant interactions between litter size and growth rate were detected using ANOVA.

### Chronic paternal ethanol exposure associates with long-term effects on glucose metabolism and insulin signaling

**Fig. 2:**
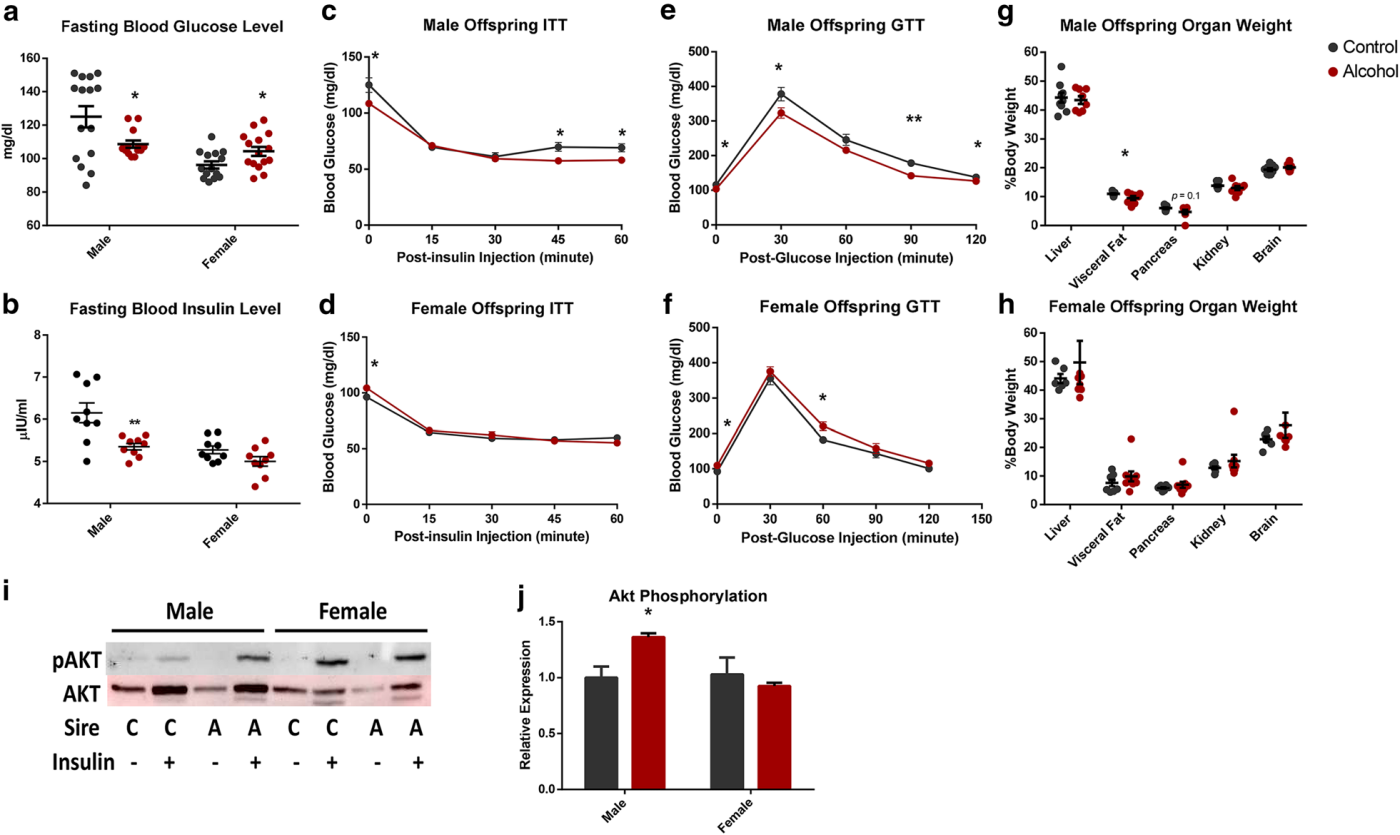
Chronic preconception male ethanol exposure exerts sex-specific effects on offspring metabolic function. **a** Fasting blood glucose levels compared between preconception treatments (*n* = 15 males, 15 females). **b** Fasting insulin levels compared between preconception treatments (*n* = nine males, nine females). **c**–**f** Glucose tolerance (GTT) and insulin tolerance (ITT) tests in the offspring of ethanol-exposed and control males (*n* = 15 males, 15 females). Organ weights of **g** male and **h** female offspring compared between the two preconception treatment groups (*n* = nine males, nine females). **i** Representative immunoblot comparing total and phosphorylated AKT (Ser473) between male and female offspring sired by ethanol-exposed and control males (*n* = six males and six females). **j** Densitometry analysis of immunoblots comparing total and phosphorylated AKT (*n* = six males and six females). Data analyzed using either an unpaired *t* test or a two-way ANOVA followed by Sidak post hoc analysis. Error bars represent SEM *p < 0.05 and **p < 0.01 (comparisons between alcohol and control preconception treatments)

In clinical studies, IUGR children develop impaired insulin responses to glucose and similar observations have been reported in both sheep and rat models of fetal growth restriction [105–108]. To determine whether the IUGR observed in the offspring of ethanol-exposed males impacted the long-term regulation of blood sugar homeostasis, glucose and insulin stress tests were conducted. Paternal ethanol consumption associated with a significant decrease in both fasting blood glucose and insulin levels in male offspring, while female offspring displayed an increase in fasting glucose levels only (Fig. 2a, b). At 8 weeks of age, these alterations were associated with exaggerated insulin responses in both glucose and insulin tolerance tests within the male offspring, while females displayed a modest impairment in their glucose tolerance test (Fig. 2c–f). To identify the pathophysiological basis of these altered parameters, mice were killed at 8 weeks of age, their organs weighed and tissues collected for molecular analysis. The male offspring of ethanol-exposed sires displayed a 13% reduction in visceral fat (*p* = 0.04), and while pancreas weight tended to be smaller (*p* = 0.1); this did not reach statistical significance (Fig. 2g). No differences in organ weights were noted in the female offspring (Fig. 2h).

To determine whether preconception alcohol exposure impacted the insulin signaling pathway, six randomly selected, eight-week-old male and female mice from each preconception treatment group had their livers perfused with insulin to assay *Akt* signaling. *AKT* phosphorylation (Ser473) in the insulin-perfused livers was increased by 26% in the male offspring of ethanol-exposed sires (*p* = 0.014), while female offspring were identical to the controls (Fig. 2i, j). Collectively, these observations indicate preconception male alcohol exposure is associated with programmed metabolic dysfunction, and is similar to data reported in long-term clinical studies of FASD children, in which disruptions in the endocrine regulation of blood glucose levels have been observed [6].

### Preconception paternal alcohol exposure associates with markers of hepatic fibrosis in the adult offspring

**Fig. 3:**
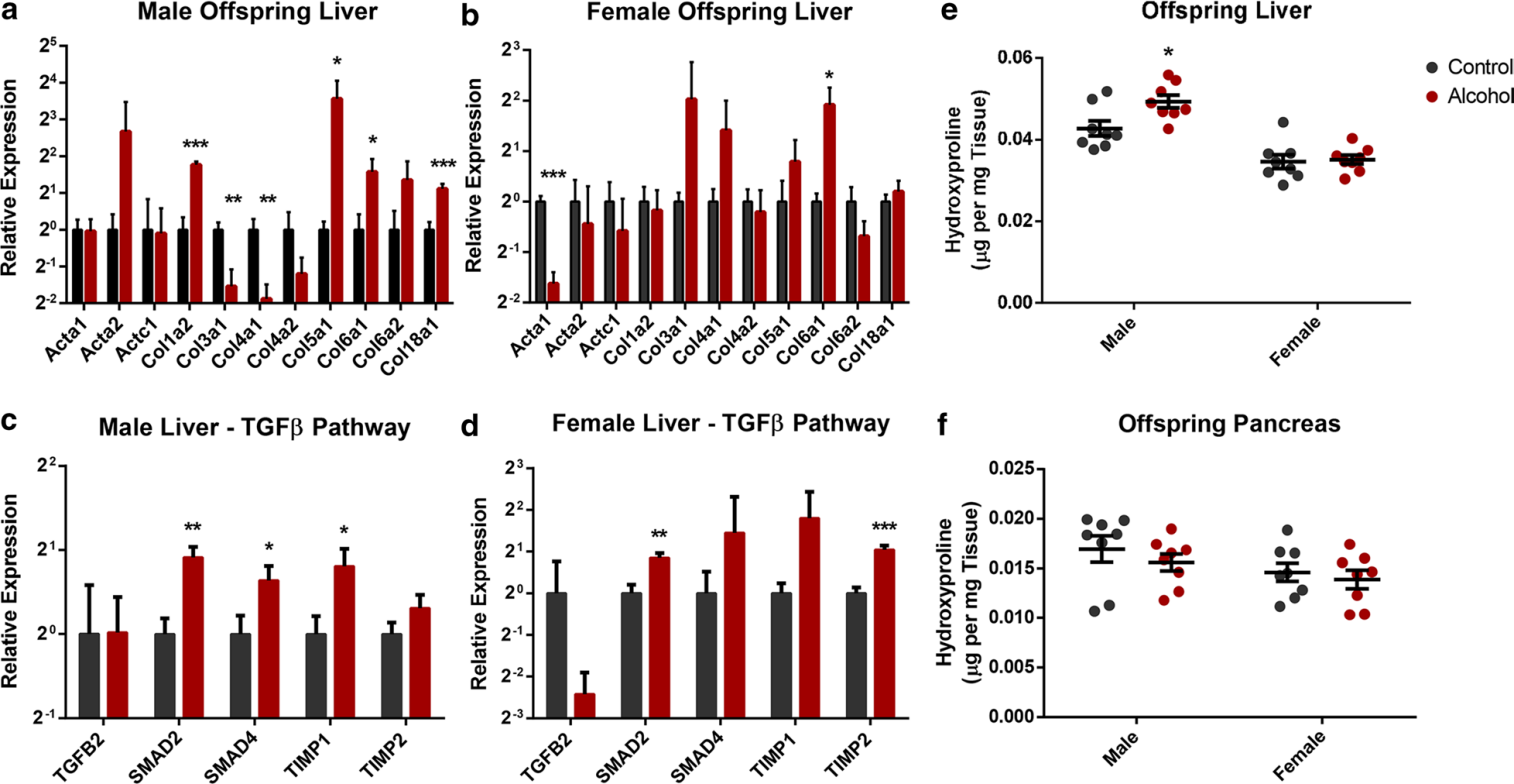
Preconception paternal ethanol exposure imparts a legacy of increased hepatic fibrosis within the male offspring. RT-qPCR analysis of genes encoding pro-fibrotic structural proteins in the adult liver of **a** male and **b** female offspring (*n* = 8). RT-qPCR analysis of genes within the pro-fibrotic TGF-B signaling pathway in the livers of 8-week-old **c** male and **d** female offspring sired by ethanol-exposed and control males (*n* = 8). Total levels of cellular hydroxyproline within the **e** livers and **f** pancreas of 8-week-old offspring sired by ethanol-exposed and control males (*n* = 8). For RT-qPCR analyses, measured Ct values were normalized to the geometric mean of transcripts encoding *Ywhaz*, *Hprt*, and *Mrpl1*, and graphed relative to the control treatment. Graphs represent independent replicates, with two independent RT reactions and three RT-qPCR measurements for each RT. Data analyzed using either an unpaired *t* test or a two-way ANOVA followed by Sidak post hoc analysis. Error bars represent SEM *p < 0.05, **p < 0.01 and ***p < 0.001 (comparisons between alcohol and control preconception treatments)

In previous studies, intrauterine growth restriction has been associated with alterations in offspring blood lipid profiles and the development of adolescent non-alcoholic fatty liver disease (NAFLD) [19, 109]. In our previous studies examining the fetal development of offspring sired by ethanol-exposed males, we observed alterations in placental cholesterol transport and the emergence of molecular markers associated with hepatic fibrosis [58]. To determine whether the legacy of preconception ethanol exposure previously identified in the fetal liver extends into postnatal life, the expression of the genes identified in the transcriptomic analysis of the fetal liver were examined by RT-qPCR [58]. In male offspring, and to a lesser extent females, preconception paternal ethanol exposure continued to associate with persistent alterations in the expression of multiple collagen subtypes and core components of the *TGF*-*β* signaling pathway driving hepatic fibrosis [110], even at 8 weeks of age (Fig. 3a–d). To determine if this transcriptional signature associated with increased molecular markers of hepatic fibrosis, we measured total levels of hydroxyproline, a commonly employed biomarker of this condition [111]. Given the trend toward a reduction in weight (Fig. 2g), we also assayed this marker in the pancreas. The male offspring of ethanol-exposed sires displayed a 13% increase in hydroxyproline levels within the liver (*p* < 0.01), while levels of hepatic hydroxyproline in the female offspring were identical to the controls (Fig. 3e). No differences in levels of hydroxyproline content could be detected in the pancreas in either males or females (Fig. 3f). These results indicate that the molecular signature previously identified in the fetal liver [58] persists into postnatal life and suggests the existence of a heightened fibrotic response within the livers of male offspring sired by alcohol-exposed fathers.

### Preconception paternal alcohol exposure associates with disruptions in hepatic gene expression within the adult offspring

**Fig. 4:**
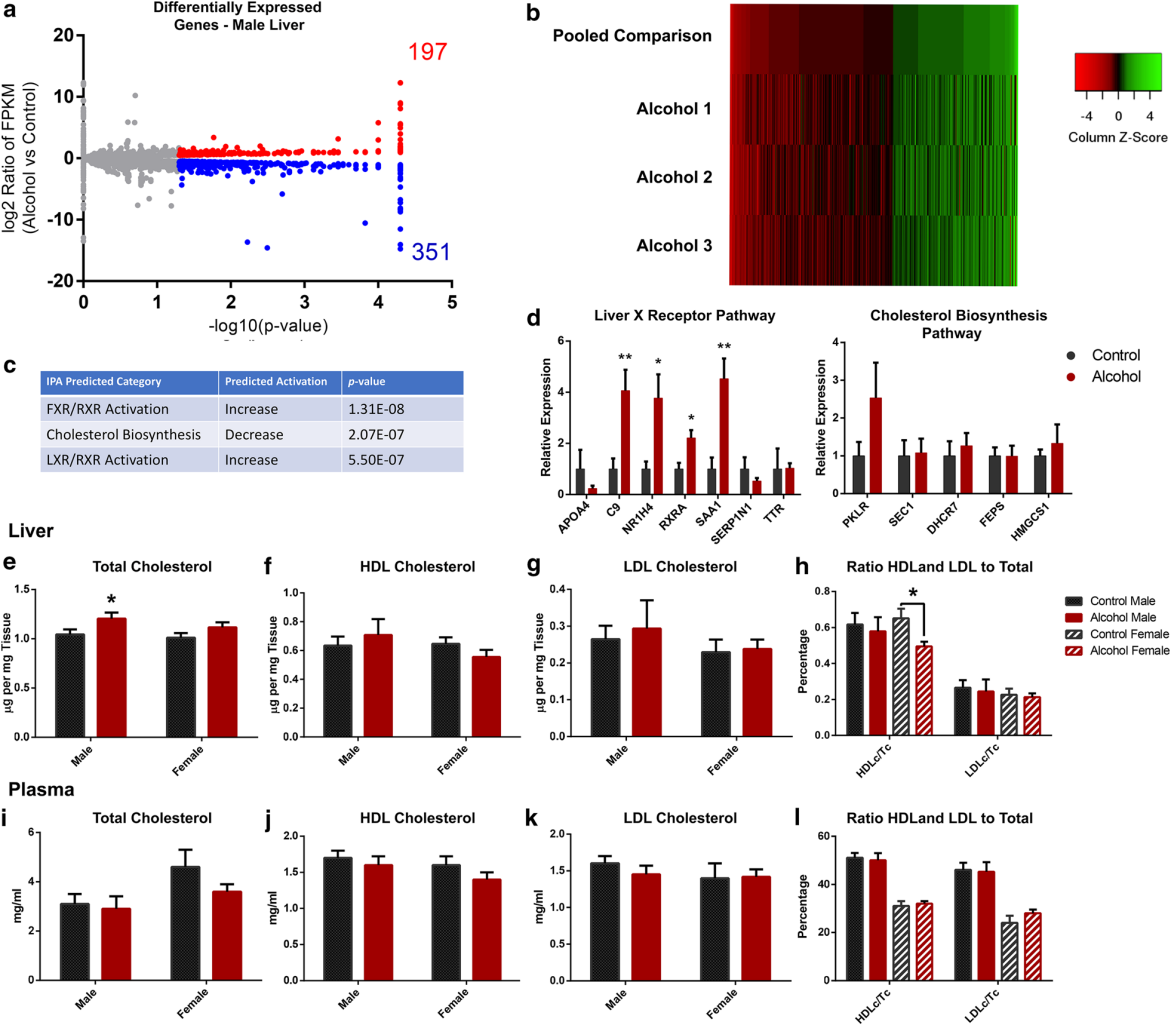
Comparison of the hepatic transcriptome between the adult offspring of ethanol-exposed and control males. **a** Volcano plot displaying differences in gene expression between adult male offspring sired by ethanol-exposed and control fathers (*n* = 3, cutoff q = 0.05). **b** Heatmap depicting the differentially expressed genes between the pooled control offspring and each of the three offspring of the alcohol-exposed males. **c** Top three categories identified using Ingenuity Pathway Analysis. **d** RT-qPCR validation of candidate genes related to the identified FXR/RXR, FXR/RXR and cholesterol biosynthesis pathways in the adult male liver (*n* = 8). Levels of hepatic **e** total cholesterol, **f** high-density lipoprotein and **g** low-density lipoprotein cholesterol esters within the liver of male and female offspring sired by ethanol-exposed and control fathers (*n* = 8). **h** Proportional ratios of HDL cholesterol to total cholesterol and LDL cholesterol to total cholesterol in liver samples derived from the male and female offspring of ethanol-exposed and control males (*n* = 8). Quantification of **i** total cholesterol, **j** high-density lipoprotein and **k** low-density lipoprotein cholesterol esters within the plasma of male and female offspring sired by ethanol-exposed and control fathers (*n* = 8). **l** Proportional ratios of HDL cholesterol to total cholesterol and LDL cholesterol to total cholesterol in plasma derived from the male and female offspring of ethanol-exposed and control males (*n* = 8). For RT-qPCR analyses, measured Ct values were normalized to the geometric mean of transcripts encoding *Ywhaz*, *Hprt* and *Mrpl1*, and graphed relative to the control treatment. Graphs represent independent replicates, with two independent RT reactions and three RT-qPCR measurements for each RT. Data were analyzed using either an unpaired *t* test, arcsine transformed and an unpaired *t* test with Welch’s correction applied or used in a two-way ANOVA followed by Sidak post hoc analysis. Error bars represent SEM *p < 0.05 and **p < 0.01 (comparisons between alcohol and control preconception treatments)

Given the persistent changes in gene expression identified between the fetal [58] and adult liver (Fig. 3), we next assayed genome-wide patterns of transcription in the adult liver of male offspring using deep sequencing. These analyses identified 548 differentially expressed genes, with 351 down-regulated and 197 up-regulated transcripts identified (*q* = 0.05; Fig. 4a). In these analyses, we observed a high level of variability, where not all of the differentially expressed genes were consistent between the between the offspring of alcohol-exposed sires (Fig. 4b). Using Ingenuity Pathway Analysis, we identified up-regulation of candidate genes participating in the genetic pathways regulating both *LXR/RXR* and *FXR/RXR* activation, as well as down-regulation of genes participating in the cholesterol super-pathway of biosynthesis (Fig. 4c). Although we were able to validate multiple candidate genes participating in *LXR/RXR* and *FXR/RXR* signaling pathways, identified candidate genes participating in pathways regulating cholesterol biosynthesis were not significantly different (Fig. 4d). However, we did observe a 15% increase in the levels of total cholesterol in the livers of the male offspring of alcohol-exposed sires (*p* = 0.04, Fig. 4e). These differences were not linked to specific increases in either high-density or low-density lipoproteins, and no differences in their proportional relationship could be detected (Fig. 4f–h). In contrast to males, female offspring of alcohol-exposed sires displayed a 23% reduction in the proportion of high-density lipoproteins to total cholesterol, as compared to the female offspring of control fathers (*p* = 0.03, Fig. 4h). No differences in total cholesterol, high-density and low-density lipoproteins or their proportional relationships were observed within the adult plasma for either sex (Fig. 4i–l).

### Preconception paternal alcohol exposure associates with long-term alterations in immune signaling within the offspring

**Fig. 5:**
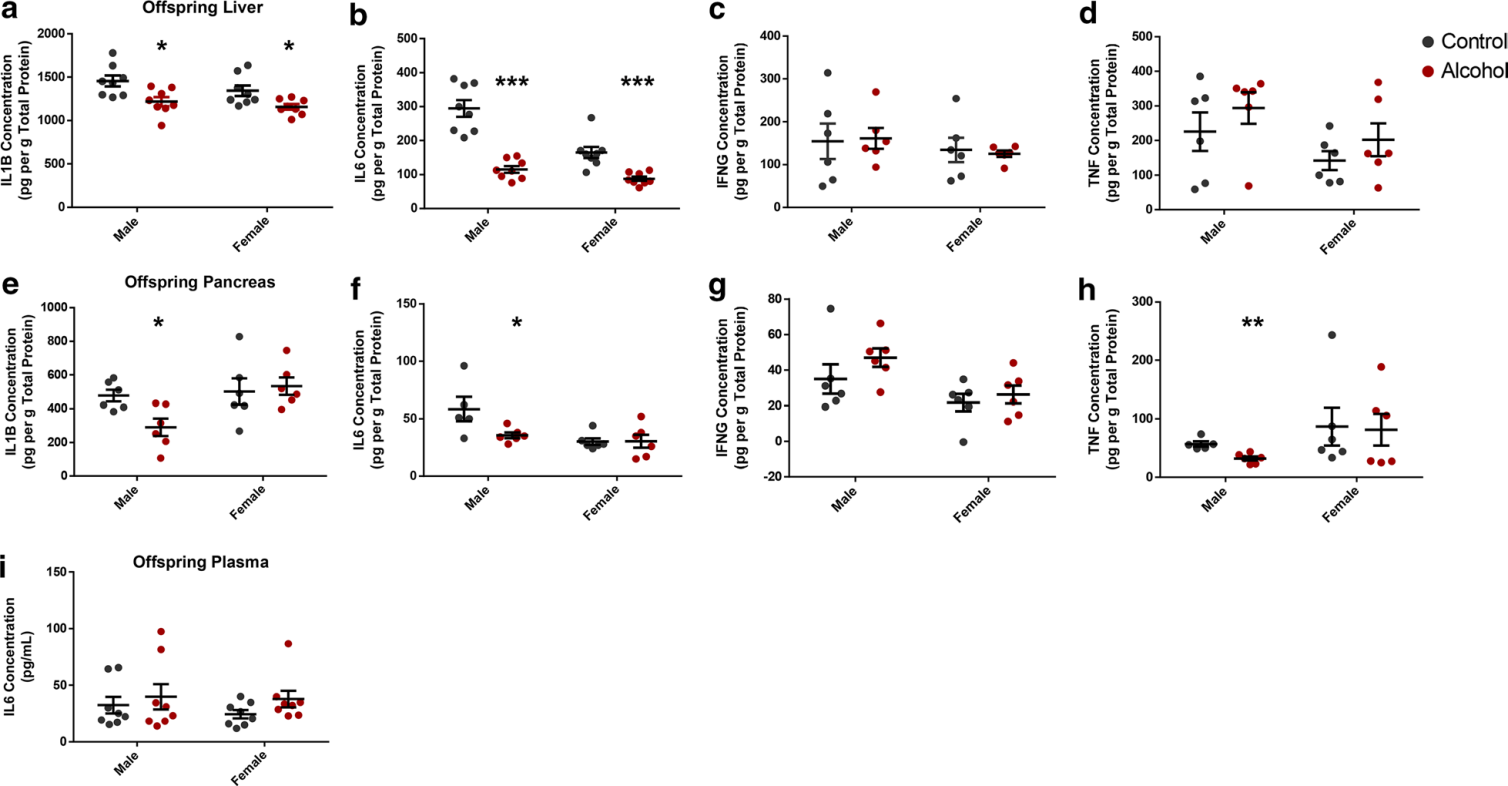
Preconception paternal alcohol exposure associates with long-term alterations in immune signaling within the offspring. Abundance of the inflammatory cytokines *IL1B*, *IL6, INFg* and *TNFa* in the **a**–**d** liver, **e**–**h** pancreas and **i** plasma of 8-week-old offspring sired by ethanol-exposed and control males (*n* = 8). Data analyzed using a two-way ANOVA followed by Sidak post hoc analysis. Error bars represent SEM *p < 0.05, **p < 0.01 and ***p < 0.001 (comparisons between alcohol and control preconception treatments)

Clinical studies of small-for-gestational-age neonates have associated alterations in key inflammatory markers with increased hepatic fibrosis [112, 113]. However, the mechanistic basis underlying these abnormalities has been challenging to decipher. *Liver X receptors* (*LXRs*) are known to modulate numerous aspects of hepatic cholesterol metabolism but have also been found to modulate immune and inflammatory responses in tissue resident macrophages [114]. Specifically, activation of *LXR/RXR* and *FXR/RXR* pathways suppresses tissue inflammatory responses via *NFK*-*Β* signaling and block the downstream release of multiple cytokine signaling molecules [115]. We, therefore, assayed the liver, pancreas and plasma for alterations in *IL1Β*, *IL6*, *INFγ* and *TNFα*, which are all established markers of inflammation linked to *LXR* and *NFK*b signaling [116, 117]. In the liver, a ~ 15% decrease in *IL1B* and a 60% decrease in *IL6* were identified in both male and female offspring, while no alterations in *INFG* and *TNFa* were observed (Fig. 5a–d). In the pancreas, the male offspring of alcohol-exposed sires displayed a ~ 40% decrease in the levels of *IL1Β*, *IL6* and *TNFα*, while levels of *INFγ* were similar to the controls (Fig. 5e–h). No differences in any of the measured cytokines were observed in the pancreas of female offspring (Fig. 5e–h). In the plasma, only *IL6* could be detected and no significant differences were observed in either males or females (Fig. 5i). These results are similar to previous clinical observations of FASD adolescents, which have also reported immune suppression within this patient group [7–9].

### Preconception alcohol exposure does not impact the regulation of imprinted genes

This study utilized two strains of mice carrying distinct single nucleotide polymorphisms within both the promoter regions and messenger RNAs of multiple genes. When using F1 hybrid crosses between the B6(CAST7) strain and a C57BL/6J strain, we can distinguish maternal and paternal alleles using C57BL/6(Cast7) and C57BL/6J polymorphisms. Using informatic approaches, we mined our RNA sequence datasets to determine if any imprinted genes exhibited abnormal bi-allelic expression (Table 1). We were able to identify exclusively paternal expression for *Peg3*, exclusively maternal expression for *Snrpn* and bi-allelic expression of *Ube3a*, which is only imprinted in the brain. None of the candidate genes examined displayed any detectable abnormalities in imprinted gene expression, and no candidate genes were identified as differentially expressed in the RNA-seq dataset.

**Table 1:** Abundance of B6(CAST7) and C57BL/6J polymorphisms identified within select imprinted genes

Gene	SNP location	C57BL/6J	Control 1	Control 2	Control 3	Alcohol 1	Alcohol 2	Alcohol 3
Ascl2	chr7:142,968,971	G	ND	ND	ND	ND	ND	ND
Cdkn1c	chr7:143,460,109	A	A/6	A/6	A/7	A/10	A/3	A/10
Dcn	No SNP found		ND	ND	ND	ND	ND	ND
Dlk1	chr12:109,460,379	A	ND	ND	A/1	ND	ND	ND
Gatm	chr2:122,594,926	C	ND	ND	ND	ND	ND	ND
Gnas	No SNP found		ND	ND	ND	ND	ND	ND
Grb10	chr11:11,954,907	A	A/1	ND	ND	ND	ND	ND
Gtl2	chr12:109,545,837	A	A/1	A/1	ND	ND	ND	ND
H19	chr7:142,577,095	T	ND	ND	T/1	T/1	T/1 C/1	ND
Igf2 (1)	chr7:142,652,037	G	ND	ND	ND	ND	ND	ND
Igf2 (2)	chr7:142,652,936	C	ND	ND	ND	ND	G/2	ND
Igf2r	chr17:12,682,709	C	C/64	C/116	C/171	C/126	C/67	C/87
Mest	chr6:30,747,382	A	A/2	ND	ND	ND	ND	A/1
Ndn	chr7:62,348,457	T	C/2	C/1	C/1	ND	ND	ND
Peg3	chr7:6,706,217	G	T/4	T/12	T/5	T/2	T/8	T/14
Sgce	chr6:4,717,926	T	T/5	T/4	T/1	T/3	ND	T/4
Slc38a4	chr15:96,995,974	C	C/89 A/1	C/78 A/1	C/26	C/117 A/1	C/74 A/1	C/72
Snrpn	chr7:59,983,415	T	T/49	T/76	T/120	T/94	T/48	T/72
Ube3a	chr7:59,228,878	T	T/7 C/5	T/8 C/8	T/3 C/5	T/16 C/10	T/7 C/9	T/6 C/3
Zac1	chr10:13,128,934	G	G/1	G/5	G/3	G/3	ND	G/1
Zim1	chr7:6,675,637	A	ND	ND	ND	ND	ND	ND

The abundance of B6(CAST7) and C57BL/6J polymorphisms within the RNA sequencing profiles of the adult liver were compared between the offspring of males exposed to the two preconception treatments

## Discussion

Our group recently reported sex-specific patterns of fetal growth restriction in a mouse model of preconception male ethanol exposure [58]. This growth restriction predominantly impacted the female offspring of ethanol-exposed sires and was accompanied by a 12% decrease in placental efficiency, abnormal placental cholesterol transport and altered markers of hepatic fibrosis within the fetal liver. The aim of the present study was to determine whether these paternally inherited abnormalities cause any long-term impacts on the growth and metabolic health of the offspring. Here, we report that chronic paternal ethanol exposure associated with a prolonged period of fetal gestation and an increased incidence of intrauterine growth restriction, which affected the male offspring to a greater extent than the females. In the male offspring, these growth deficits persisted into adult life and associated with insulin hypersensitivity, increased markers of hepatic fibrosis and alterations in immune signaling.

How preconception paternal ethanol exposure leads to the sequelae described above is not understood. We suspect that similar to other models of altered developmental programming, ethanol-induced disruptions in the sperm-inherited epigenetic program alter the formation or function of the placenta, which leads to long-term alterations in developmental programming within the offspring [26–28]. In our model, we observed a 7% decrease in the weight of only the female offspring at gestational day 14.5 [58], while 1 week after birth, both the male and female offspring display significant growth restriction (25% in males 15% in females). These observations indicate that the large majority of growth restriction predominantly occurs during the later phases of pregnancy when the mouse fetus experiences a dramatic increase in growth rate [118]. This late-phase growth restriction is similar to the phenotypes reported in studies of placental-specific *Insulin growth factor 2* (*Igf2*) loss of function, as well as prenatal nutrient restriction, which also predominantly manifests late in pregnancy [119, 120]. In these studies, the placenta initially compensates by increasing its overall efficiency through proportional increases in the labyrinthine layer (initially) and up-regulation of System A amino acid transporters (later) [120]. However, these strategies cannot sustain fetal growth through the late phases of pregnancy, when the mouse fetus is normally growing most rapidly in absolute terms [121]. In our model, we observed decreased placental efficiency at gestational day 14.5, indicating the placenta enters the late growth phase at a functional deficit [58]. We suspect that as pregnancy proceeds, this placental dysfunction progressively gets worse, ultimately causing growth restriction and the delay in partition identified in Fig. 1. Further studies examining placental morphology and function between gestational days 16 and 19 are required to define the physiological basis of this growth restriction.

Although very few clinical studies have followed FASD children into adolescence (reviewed here [10]), the few that have, report a number of observations that share many similarities with results presented in this report. For example, a recent study examining a patient cohort from Cape Town, South Africa, followed the height, weight and head circumference of alcohol-exposed babies through to 13 years of age [5]. In this cohort, alcohol-exposed children had a growth trajectory that was significantly less than the control group at all ages examined. In our model of paternal ethanol exposure, we observed significant growth restriction at 1 week of age, which in the male offspring, persisted despite an overall increase in growth rate. Although the Cape Town study did not conduct separate analyses of males and females, other studies indicate that male FASD children have lower postnatal viability compared to females, indicating FASD growth defects may impact boys to a greater extent than girls [122].

We observed insulin hypersensitivity in the male offspring of ethanol-exposed fathers, which correlated with long-term deficits in growth. Our observations indicate this increased response is due to heightened sensitivity of the hepatic insulin signaling pathway, although the specific molecular mechanisms by which this arises are still under investigation. Interestingly, our data are in direct contrast with both a clinical case report examining the metabolic health of FASD children [6], as well as work conducted using a rat model of prenatal maternal ethanol exposure, in which insulin resistance was observed [123, 124]. However, in the rat model, both the male and female F2 progeny of ethanol-exposed offspring displayed hypoglycemia and hyperinsulinemic response patterns [124]. Combined with our work, these observations suggest that ethanol can program an insulin hypersensitive phenotype, which, similar to alcohol-induced disruptions to the hypothalamic-pituitary axis [79], can transmit to the next generation via the germline.

Clinical studies have shown that, even after controlling for low maternal income, maternal smoking and birthweight, newborns whose mothers drank during pregnancy have a threefold increased risk of infection compared to mothers who did not drink [9]. FASD infants exhibit an increased risk of developing upper respiratory tract infections, recurrent otitis media, pneumonia, persistent diaper rash, meningitis and gastroenteritis [7, 8]. These deficits in immune function have been linked to alterations in the number of T helper (*CD4*+*/CD3*+) and T cytotoxic/suppressor cells (*CDS*+*/CD3*+) [8], suggesting the processes driving systemic inflammation are blunted in these children. Our studies examining the offspring of ethanol-exposed males identified alterations in hepatic *LXR* signaling, which, through the activity of *NFKb*, is a potent suppressor of multiple cytokine signaling pathways [114, 115]. Consistent with these observations, we identified tissue-specific reductions in multiple *NFKb* regulated genes (*IL1B*, *IL6* and *TNFa*) within the liver and pancreas but not in the plasma. It is unclear if the reductions in immune signaling observed in our model have any relevance to the enhanced predisposition of FASD children to postnatal infection; however, programmed alterations in systemic inflammation appear to be a common theme emerging in other models of developmental programming [125]. Additional studies are needed to determine how preconception paternal ethanol exposure influences the inflammatory stress response.

Consistent with previous studies [126], placental dysfunction occurring late in gestation predominantly affected the long-term health of the male fetus, while the female offspring were better able to recover. These observations suggest that females are better able to compensate for late-stage insults than males. Interestingly, while hepatic fibrosis is one of the defining symptoms of alcoholic liver disease in adults [127], three isolated case reports have also identified a similar condition in FASD children [128–130]. Our previous studies of the fetal liver identified alterations in the transcription of candidate genes participating in the genetic pathways regulating hepatic fibrosis and stellate cell activation [58]. Although found in both the male and female offspring of ethanol-exposed sires, this signature was diametrically opposite between the sexes; males displayed an up-regulation of pro-fibrotic genes, while females suppressed this pathway. In the liver, 72% of genes are expressed in a sexually dimorphic manner and importantly, half of the candidate gene identified in this study follow this pattern [131]. Therefore, sex-specific differences in patterns of hepatic gene expression may explain the contrasting outcomes between the male and female offspring. Importantly, the persistence of this signature in the adult male liver, along with the increased hydroxyproline content, suggests that preconception paternal alcohol exposure may predispose the offspring to hepatic dysfunction and susceptibility to liver disease. If true, this would significantly enhance our understanding of the mechanisms of inheritance at work in the development and progression of alcoholic liver disease. Additional studies are necessary to determine the importance this pro-fibrotic signature has in hepatic disease pathogenesis.

Finally, although we were unable to validate the differential expression of genes participating in the cholesterol biosynthesis pathway, we did observe a modest increase in total cholesterol levels in the male offspring of alcohol-exposed sires and a proportional reduction in the ratio of high-density lipoproteins to total cholesterol in the female offspring. The failure to identify changes in cholesterol-related transcripts is likely reflective of the variability of this model, which unlike a genetic model is more prone to produce a subset of offspring that are highly affected while others exhibit a more modest phenotype. Indeed, although select collagen subtypes were present in our RNA-seq datasets, fibrosis did not emerge as an enriched pathway, indicating this phenotype is also highly variable. Future studies will explore the impact the stress of a high-fat diet has on the offspring of alcohol-exposed fathers and will help determine both the penetrance of the metabolic phenotypes and the extent of the sexual dimorphism in this preconception model of exposure.

One potential confound to this study is the use of the 0.066% (w/v) Sweet’N Low in both the control and alcohol preconception treatments, which was used to promote the consistent consumption of ethanol [90]. The gut contains glucose receptors that are stimulated by artificial sweeteners causing the release of incretin peptide hormones, which have a significant role in glucose homeostasis, metabolic control and proper β-cell function [132]. Artificial sweeteners also influence the intestinal microbiota and alter metabolic function indirectly [95]. Therefore, it is possible that some of the effects are the result of metabolic disturbances in the sires. However, we did not observe any differences in weight gain between the preconception treatments and neither group displayed an obese phenotype. In addition, genetic differences unique to the C57BL/6(Cast7) and C57BL/6J cross may influence the observed phenotypes. However, the ability to informatically distinguish the maternal and paternal alleles allowed us to examine imprinted gene expression, which is disrupted in other models of developmental programming [133]. Future studies will focus on implementing an exposure paradigm that does not involve the use of artificial sweeteners and repeating these observations using both a pure C57BL/6J cross and an outbred line.

## Conclusions

Non-syndromic developmental defects are multifactorial and are hypothesized to result from the complex interplay between genetic, epigenetic, environmental and lifestyle factors [134, 135]. Our data further support the epidemiological association between paternal alcohol consumption and deficits in child development [33–45, 136]. Specifically, our study associates chronic preconception paternal ethanol exposure with prenatal/postnatal growth restriction, sex-specific alterations in long-term metabolic function, immune dysfunction and hepatic fibrosis. As discussed above, these pathologies have been observed in long-term studies of FASD children, indicating paternal alcohol use is a relevant factor in the etiology of this disorder. Given the fact that male alcohol consumption far exceeds that of women [92, 93], we should more carefully consider the preconception lifestyle choices of the birth-father in the development of this condition and broaden our educational outreach concerning the teratogenic actions of this agent.

## Additional files

**Additional file 1.** Primer sequences used in RT-qPCR analyses.

**Additional file 2.** Supporting information for Figure 1. A) Datasets discussed in Figure 1 are presented in table form. B) Formula used to calculate the IUGR ratio. C) Formula used to calculate growth rate of the offspring.

## Abbreviations

FASDs: fetal alcohol spectrum disorders
LXR: LiverX receptor
RXR: RetinoidX receptor
FXR: FarnesoidX receptor
IUGR: intrauterine growth restriction
AKT: protein kinase B
FPKM: fragments per kilobase of exon per million fragments mapped
qPCR: real time quantitative polymerase chain reaction
RT-qPCR: reverse transcriptase quantitative polymerase chain reaction
Ct: cycle threshold
Ywhaz: 3-monooxygenase/tryptophan 5-monooxygenase activation protein zeta
Mrpl1: mitochondrial ribosomal protein L1
Hprt: hypoxanthine phosphoribosyl transferase
NAFLD: non-alcoholic fatty liver disease
TGF-B: transforming growth factor-beta
NFkB: nuclear factor-kappa-B
IL-6: interleukin-6
IL1B: interleukin-1 beta
INF-g: interferon gamma
TNFa: tumor necrosis factor alpha
C9: complement C9
Smad2: mothers against DPP homolog 2
Smad4: mothers against DPP homolog 4
Col5a1: collagen type V alpha 1 chain
Col6a1: collagen type VI alpha 1 chain
Col18a1: collagen type XVIII alpha 1 chain
Saa1: serum amyloid A1
Nh1r4: farnesoid X-activated receptor
Peg3: paternally expressed gene 3
Snrpn: small nuclear ribonucleoprotein polypeptide N
Ube3a: ubiquitin protein ligase E3A
Igf2: insulin growth factor 2
