# Supplementary material for: Preconception paternal alcohol exposure exerts sex-specific effects on offspring growth and long-term metabolic programming
Source: Epigenetics Chromatin. 2019 Jan 22;12:9. doi: 10.1186/s13072-019-0254-0 (PMC6341619; doi:10.1186/s13072-019-0254-0)
Supplement: Supplementary file 2 — Additional file 2. Supporting information for Figure 1. A) Datasets discussed in Figure 1 are presented in table form. B) Formula used to calculate the IUGR ratio. C) Formula used to calculate growth rate of the offspring. [file 13072_2019_254_MOESM2_ESM.pdf]

A.

| | Control (Mean ± SEM) | Alcohol (Mean ± SEM) |
|---|---|---|
| Plasma Alcohol of Sires Day 10 (mg/dl) | 1.23 ± 0.04 (n = 5) | 178.31 ± 7.89 (n = 6) |
| Plasma Alcohol of Sires Day 70 (mg/dl) | 1.50 ± 0.04 (n = 5) | 245.05 ± 13.46 (n = 6) |
| Body Weight of Sires (g) | 26.58 ± 1.32 (n = 5) | 25.85 ± 0.93 (n = 6) |
| Gestation Length (Days) | 21.25 ± 0.25 (n = 4) | 23.00 ± 0.41 (n=4) |
| Litter Size | 7.20 ± 0.48 (n = 15) | 6.84 ± 0.48 (n = 19) |
| IUGR Ratio | 0.00 ± 0.00 (n = 15) | 0.05 ± 0.02 (n = 19) |
| Male Growth Rate | 5.95 ± 0.35 (n = 15) | 7.80 ± 0.51 (n = 15) |
| Female Growth Rate | 4.39 ± 0.17 (n = 15) | 5.41 ± 0.23 (n = 15) |

B.

IUGR Definition: Fetal Weight < (Mean of Control Fetal Weight – 2 X Control Fetal Weight SD)

IUGR Ratio Equation: $\text{IUGR Ratio} = \frac{\text{IUGR Fetus}}{\text{Total Fetus}}$

C.

Growth Rate Equation: $\frac{\text{Body Weight W8} - \text{Body Weight W1}}{\text{Body Weight W1}}$
